# DAILY SOCIAL INTERACTIONS AND BIOLOGICAL STRESS REACTIVITY: ARE THERE AGE DIFFERENCES?

**DOI:** 10.1093/geroni/igz038.2717

**Published:** 2019-11-08

**Authors:** Courtney A Polenick, Steven H Zarit, Kira S Birditt

**Affiliations:** 1 University of Michigan, Ann Arbor, Michigan, United States; 2 Pennsylvania State University, University Park, Pennsylvania, United States

## Abstract

Older people experience fewer negative social interactions and report less anger and stress when faced with interpersonal tensions. Little is known, however, about age differences in biological responses to social interactions. We evaluated how salivary DHEA-S, a key indicator of stress reactivity, is associated with daily positive and negative social interactions among midlife and older adults. Participants were drawn from the Daily Health, Stress, and Relationship Study, which includes 93 adults age 40 to 95 who completed 14 days of daily diary interviews and provided saliva samples on four of those days. Multilevel models showed that people had higher DHEA-S on days in which they reported more positive interactions. Older respondents were less reactive to negative interactions relative to younger respondents. These findings indicate that positive social interactions may benefit biological stress reactivity regardless of age, whereas older adults are more resilient to the adverse effects of negative social interactions.

